# Photon counting-energy integrating hybrid flat panel detector systems for image-guided interventions: an experimental proof-of-concept

**DOI:** 10.1088/1361-6560/acddc7

**Published:** 2023-06-28

**Authors:** Kevin Treb, York Hämisch, Christer Ullberg, Ran Zhang, Ke Li

**Affiliations:** 1 Department of Medical Physics, School of Medicine and Public Health, University of Wisconsin-Madison, 1111 Highland Avenue, Madison, WI, United States of America; 2 Direct Conversion GmbH, Lochhamer Schlag 10, D-82166 Graefelfing, Germany; 3 Varex Imaging, Svärdvägen 23, S-182 33 Danderyd, Sweden; 4 Department of Radiology, School of Medicine and Public Health, University of Wisconsin-Madison, 600 Highland Avenue, Madison, WI, United States of America

**Keywords:** photon counting imaging, photon counting CT, x-ray detector, C-arm interventional x-ray systems, cone-beam CT

## Abstract

*Objective.* Current C-arm x-ray systems equipped with scintillator-based flat panel detectors (FPDs) lack sufficient low-contrast detectability and spectral, high-resolution capabilities much desired for certain interventional procedures. Semiconductor-based direct-conversion photon counting detectors (PCDs) offer these imaging capabilities, although the cost of full field-of-view (FOV) PCD is still too high at the moment. The purpose of this work was to present a hybrid photon counting-energy integrating FPD design as a cost-effective solution to high-quality interventional imaging. *Approach.* In the proposed hybrid detector design, the central scintillator and thin-film transistor elements in the FPD are replaced with a semiconductor PCD module to upgrade the imaging capabilities of the C-arm system while preserving the full FOV coverage. The central PCD module can be used for high-quality 2D and 3D region-of-interest imaging with improved spatial- and temporal-resolution as well as spectral resolving capability. An experimental proof-of-concept was conducted using a 30 × 2.5 cm^2^ CdTe PCD and a 40 × 30 cm^2^ CsI(Tl)-aSi(H) FPD. *Main results.* Phantom and *in vivo* animal studies show (1) improved visualization of small stent wires in both 2D and 3D images due to the better spatial resolution of the PCD; (2) dual-energy angiography imaging capability by using the spectral PCD; (3) better conspicuity of small peripheral iodinated vessels (contrast-to-noise ratio improvement range: (29%, 151%)); (4) the central PCD outputs can be fused seamlessly with the surrounding scintillator detector outputs to provide full field imaging: A post-processing chain was developed by leveraging the PCD’s spectral information to match the image contrast of the PCD images to the surrounding scintillator detector, followed by spatial filtering of the PCD image to match noise texture and spatial resolution. *Significance.* The hybrid FPD design provides a cost-effective option to upgrade C-arm systems with spectral and ultra-high resolution capabilities without interfering with the clinical need for full FOV imaging.

## 1. Introduction

Minimally-invasive image-guided interventions (IGIs) are important components in the armamentarium of disease treatment, including for acute ischemic stroke (AIS) due to large vessel occlusion, hepatocellular carcinoma (HCC), etc. C-arm x-ray systems equipped with digital flat panel detectors (FPDs) are a mainstay in IGIs due to their real-time x-ray image-guidance capabilities such as fluoroscopy and digital subtraction angiography (DSA), as well as cone-beam CT (CBCT). The success of interventional procedures and clinical outcomes relies heavily on the quality and utility of the images that can be obtained in the interventional room. For example, if an intracranial hemorrhage (ICH) during endovascular thrombectomy for AIS can be recognized timely for immediate complication management, morbidity and mortality can be significantly reduced (Patel *et al* 2019). Currently, ICH is usually evaluated by transferring patients from the interventional room to a diagnostic MDCT or MRI suite due to the imaging limitations of C-arm interventional systems, namely: (1) limited low-contrast visualization in FPD-CBCT images compared to MDCT, and (2) lack of dual-energy CT capabilities in the interventional suite, for example to discriminate between iodine staining and true ICH. Although there are currently some interventional rooms equipped with both a C-arm and a sliding-gantry MDCT so that additional patient transport is not required, the wide clinical adoption of such systems is limited due to a larger required room space and the increased cost of the dedicated MDCT gantry. In addition to stroke treatment, high-quality intraoperative imaging is also desirable for ablative treatment of HCC: insufficient ablative margins are the single biggest predictor of local tumor progression (Kim *et al* 2006, Nakazawa *et al* 2007, sun Kim *et al* 2010, Teng *et al* 2015, Yoon *et al* 2018), and have low-contrast that may be difficult to resolve with CBCT. Additionally, if high-quality iodine material images are available in the interventional room immediately after ablation therapy to better detect and characterize focal hypervascularities within and at the rim of the ablation zone (Agrawal *et al* 2014, Vandenbroucke *et al* 2015), physicians can better determine whether additional ablations need to be performed to achieve a complete ablation with sufficient safety margins.

The imaging capability and performance of existing C-arm systems are strongly limited by the existing scintillator-based, energy integrating FPDs. The long transmission distances of analog signals in the a-Si(H) thin-film transistor (TFT) array and the passive pixel design results in pronounced electronic noise in the detector (Koniczek *et al* 2020); the down-weight of low-energy x-ray photons in energy integrating detectors is highly suboptimal for imaging iodine; the afterglow of scinitllator and the slow charge carrier mobilities in the a-Si impose a fundamental limit to the achievable temporal resolution and introduce undesirable lag effects; the light spatial spreading in the scintillator imposes a fundamental limit to the achievable spatial resolution. This limits the detector’s ability to resolve fine structures such as stent kinking and narrowing and corresponding vessel stenosis during IGIs; there is also a strong tradeoff between spatial resolution and scintillator thickness, which is currently only around 600 *μ*m (only 30% of the scinitllator thickness in MDCT detectors) and yields a low x-ray absorption efficiency. Lastly, the existing clinical FPDs do not provide energy resolving capability for dual-energy imaging applications.

Towards implementing dual-energy imaging capabilities to C-arm systems, a non-detector-based approach is to implement slow or fast-kV switching between x-ray beams with higher (e.g. 120 kV) and lower (e.g. 60 kV) tube potentials (Müller *et al* 2016, Speidel *et al* 2019, Nikolau *et al* 2022). This approach has demonstrated potential for dual-energy subtraction angiography (DESA) as an alternative to traditional DSA to mitigate misregistration artifacts caused by the subtraction of the early mask image from the later contrast-enhanced images. However, such techniques can still result in residual misregistration artifacts due to the temporal separation of the low- and high-kV pulses. More recent technological and engineering advances have resulted in increased interest in detector-based solutions for spectral x-ray and CBCT imaging, namely dual-layer FPDs (Lu *et al* 2019, Ma *et al* 2020, Shi *et al* 2020a, 2020b) and, more recently, photon counting detectors (PCDs).

Semiconductor-based PCDs are rapidly emerging x-ray detectors for medical imaging applications specifically for diagnostic CT imaging, and typically consist of a direct-conversion semiconductor sensor with application-specific integrated circuits (ASICs) to record the number of x-ray interactions in the sensor and to sort each interaction into energy bins based on their recorded energy. These energy bins allow for spatially- and temporally-registered spectral data to be obtained, which can potentially be leveraged for DESA and spectral CT imaging (McCollough *et al* 2020). Even for non-spectral interventional imaging, the use of energy thresholding allows for the complete rejection of electronic noise in PCDs for improved low-contrast visualization. Additionally, PCDs weight all individual detected photons equally regardless of their energy since each photon is recorded as a single count, which is a more optimal weighting for image contrast than that of energy integrating detectors (EIDs). PCDs also have negligible detector lag and ghosting which can enable the use of very high frame rates compared to EIDs, and can remove lag artifacts in CT images compared to EID-CBCT (Treb *et al* 2022). Lastly, PCDs offer the additional benefit of superior spatial resolution potential and a much more relaxed tradeoff between spatial resolution and sensor thickness through the use of a semiconductor sensor rather than a scintillator, which reduces the problem of spatial spreading of secondary light quanta in the sensor and opens up possibilities for ultra-high-resolution (UHR) x-ray imaging without sacrificing dose efficiency.

Prior works have investigated the use of PCDs to improve the imaging utility of C-arms for interventional imaging tasks: A PCD capable of a high frame rate of 1000 Hz has been investigated for high-speed angiography applications including quantification of blood velocity (Krebs *et al* 2019, Nagesh *et al* 2022a, 2022b, Troville *et al* 2022, Williams *et al* 2022, Wu *et al* 2022). Other works have directly implemented a PCD with a clinical C-arm gantry: One approach was to piggyback mount a PCD in front to the existing FPD (Ahmad *et al* 2016, Müller *et al* 2016, Ahmad *et al* 2017) for dual-energy 2D and 3D imaging with a full-width beam collimation. Another approach is to use an inverse geometry system with a smaller detector and a narrow-beam collimation (Slagowski 2017), although inverse geometry C-arms are not commercially available. Alternatively, a PCD strip was mounted on a C-arm to demonstrate the benefits of PCD-CT in improving the low-contrast detectability and spatial resolution (Ji *et al* 2021, Treb *et al* 2021, 2022). To preserve the functionality of the existing FPD, the PCD and the FPD are interchangeable. However, the current implementation requires the PCD to be mechanically translated into/out of the field-of-view (FOV) to switch between the two detector systems, which is undesirable for eventual clinical use.

This work presents a hybrid FPD design that integrates a central PCD module with a scintillator-based EID flat panel module: the scintillator/TFTs in the central region of the EID are replaced with a PCD module to form a single piece for a PCD-EID detector assembly, such that a C-arm interventional system becomes capable of full axial FOV PCD-CT as well as 2D region-of-interest (ROI) and 3D volume-of-interest (VOI) PCD imaging. The new hybrid PCD-EID flat panel design aims to preserve the functionality of the existing FPD while adding the benefits of a PCD and reducing the overall system cost compared to a design that completely replaces the EID with an equal-sized PCD. When the full FOV of the FPD along both the axial and *z* directions is required for global imaging sequences, the central PCD module output can be post-processed to match the image quality and characteristics of the surrounding EID such that it forms a seamless whole image. The purpose of this work was to experimentally demonstrate the feasibility of integrating PCD images with EID images as well as the potential benefits of the added PCD module in the integrated detector design for IGIs.

## 2. Experimental materials and methods

**Figure 1.**
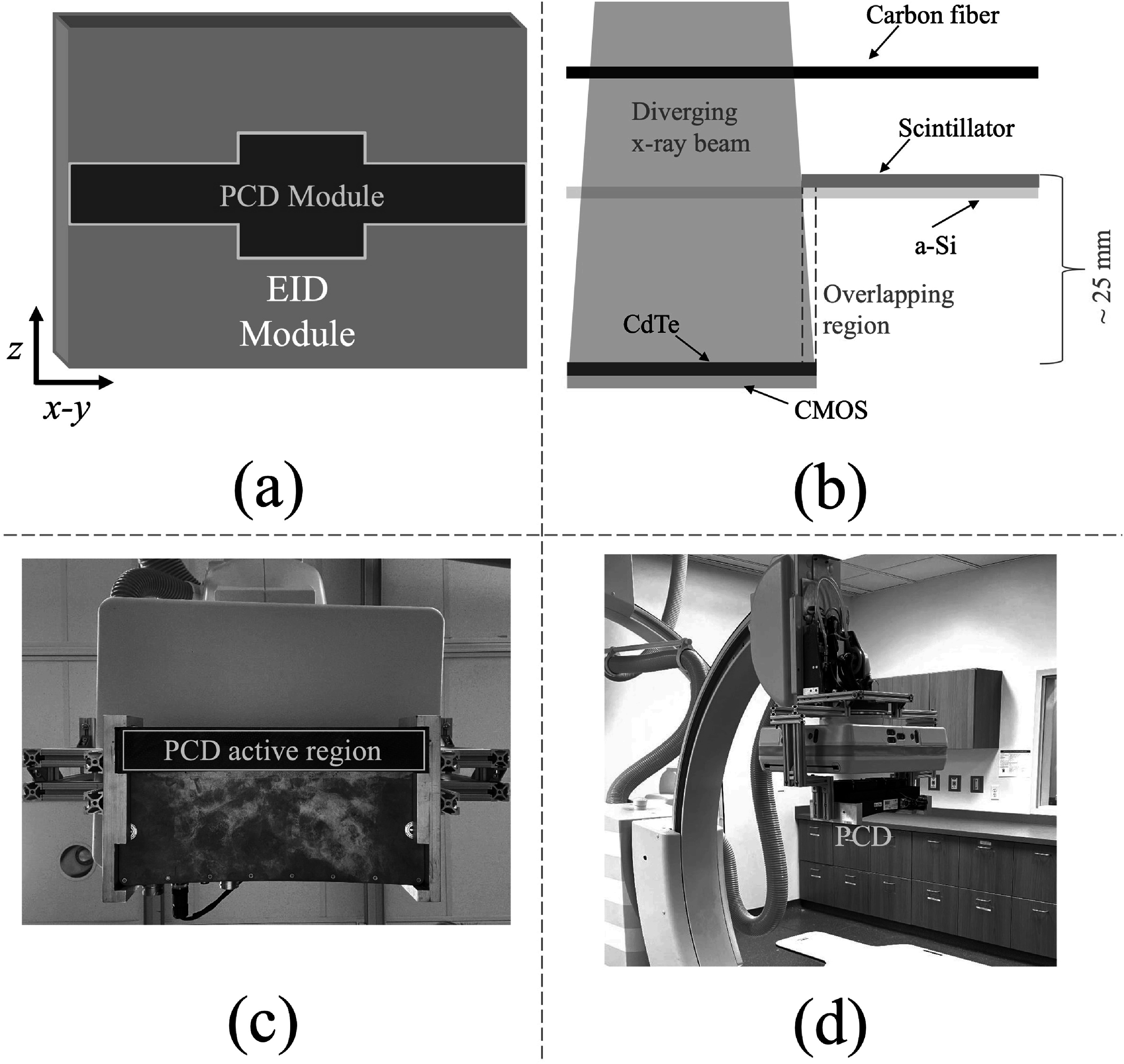
Schematics of the proposed PCD-EID flat panel design in (a) front view demonstrating a full-width PCD module for CBCT imaging with a wider center for a wider 2D imaging ROI, and (b) cross-section illustration showing the overlap between PCD and EID modules to avoid image gaps with a diverging x-ray beam (not to scale). (c)-(d) The prototype imaging system for acquiring PCD data, shown from front and side views. CMOS: complementary metal oxide semiconductors.

Shown in the schematic illustration in figure 1(a), the proposed PCD-EID flat panel includes a central PCD module with full axial coverage for spectral, low-contrast, and UHR 3D imaging as well as 2D central ROI dual-energy and dose-efficient UHR imaging. Locations of the VOI and ROI for higher-quality PCD imaging can be selected by the treating physicians based on standard full FOV fluoroscopic images or EID-CBCT. The central PCD module can be positioned such that the front face of its sensor is behind the plane of the a-Si TFT array of the surrounding EID (figure 1(b)), resulting in a slight magnification difference between the PCD and EID; this way, the useful FOV of the PCD is defined by the size and shape of the “window” in the EID where there is no scintillator material blocking the PCD surface from x-rays, and the active PCD area can extend slightly beyond this useful FOV to avoid gaps between the PCD and EID: The overlapping region in figure 1(b) would only need to be a few mm to avoid gaps between the PCD and EID due to the diverging x-ray beam projecting to a larger width on the PCD surface from magnification differences between PCD and EID. Due to differences in image quality between the central PCD and the surrounding EID, the PCD images can potentially be post-processed, as described later in section 2.3, to better match the image quality of the EID when full FOV global imaging is needed rather than high-quality ROI or VOI PCD imaging. This way the PCD-EID flat panel can be used in the same manner and with equivalent image quality as EIDs used in current clinical practice for less demanding imaging sequences such as general fluoroscopy.

As a proof-of-concept for the proposed hybrid PCD-EID design, a 30 cm × 2.5 cm PCD as well as a 40 cm × 30 cm CsI(Tl)-aSi(H)-based EID were used to separately acquire images. Table 1 summarizes other important detector specifications. Note that the thickness of the CdTe sensor of the PCD is only 0.75 mm, which is thinner than a typical sensor of around 1.6–2 mm and may result in suboptimal detection efficiency: This relatively thin sensor was chosen to have comparable x-ray absorption efficiencies between the CdTe-based PCD and the CsI-based EID (0.6 mm CsI thickness) at typical diagnostic x-ray energies. The PCD can be operated either with or without charge sharing correction (CSC) provided by the vendor (Ullberg *et al* 2013, 2018, Ji *et al* 2021). No pulse pile-up corrections were applied. The energy thresholds of the PCD were calibrated using the in-house methods described in previous work (Ge *et al* 2017) which utilizes K-edge energies for calibration. The intra- and inter-panel detector response of the PCD was calibrated using acrylic and aluminum plates of different thicknesses to correct for detector response inhomogeneities, and an in-house ring and band artifact correction was applied to PCD-CBCT (Feng *et al* 2021). The experimental system used in this work includes a rotating-anode diagnostic x-ray tube (G1592, Varex Imaging) operated under a pulsed x-ray mode for all acquisitions. Each PCD or EID image frame was synchronized with an x-ray pulse by feeding the x-ray-on signal from the high voltage generator to the trigger input of each detector. Note that the source-to-detector distance (SDD) differed between the PCD and EID acquisitions since the PCD needed to be mounted in front of the EID for the preliminary experimental studies: the measured SDDs for the PCD and EID were 106 cm and 120 cm, respectively.

**Table 1.** Specifications of the PCD and the EID used in experimental studies.

Parameters	PCD	EID
Manufacturer	Varex imaging	Varex imaging
Model number	Thor CT30	4030CB
Dimensions (cm × cm)	30 × 2.5	40 × 30
Conversion mechanism	Direct	Indirect
Sensor material	CdTe	CsI(Tl)
Sensor thickness (mm)	0.75	0.60
Pixel pitch (*μ*m)	100	194
Fill factor	100%	70%
# Energy thresholds	2	N/A
Counter bit depth (bits)	12	14

### 2.1. 2D PCD imaging studies

To demonstrate the benefits of the PCD module for 2D ROI UHR imaging, a 3 mm renal/biliary stent (Monorail 5F, Express SD, Boston Scientific, Marlborough, MA) was imaged by both the PCD and EID. The stent contained a “kinked” section where its lumen was narrowed, putting a stricter spatial resolution requirement to resolve stent wires in this section. To demonstrate the potential of the PCD for DESA, a 3 mm diameter vessel phantom containing 75 mg ml^−1^ iodine was embedded in an *ex vivo* oxtail specimen containing muscle and fat tissue and was imaged by the PCD. For all 2D PCD acquisitions, the PCD was operated with CSC turned on, and the low and high energy thresholds of the PCD were set to 15 and 63 keV respectively for the DESA acquisition: The low threshold was optimized in previous work to reject the influence of electronic noise (Ji *et al* 2019), and the high threshold was empirically determined to obtain approximately the same number of photon counts in the high- and low-energy bins. The post-log high-energy (HE) and low-energy (LE) PCD images were combined via an empirical weighted sum to remove the background tissue signal and produce a static DESA frame. More specifically for the tissue cancellation done in this work, a weighting factor *w* was chosen as the average ratio of image pixel values containing pure tissue in the post-log LE image to corresponding values in the post-log HE images such that when they are combined as

\begin{eqnarray*}\mathrm{DESA}=\mathrm{LE}-w\times \mathrm{HE},\end{eqnarray*}

where LE and HE denote post-log images, then the signal from the chosen material is canceled in the resulting DESA image, but signals from materials with different energy-dependent attenuation properties from tissue, such as iodine, remain. In this work, *w* = 1.4 was empirically chosen.

The DESA image was filtered by a Gaussian kernel with a standard deviation of 150 *μ*m for noise reduction. 2D projection images were acquired at 120 kV with air Kerma level of 0.11 *μ*Gy for the stent images and 11 *μ*Gy for the DESA vessel phantom images; all 2D acquisitions in this work used an x-ray flux rate of approximately 9 × 10^6^ counts per second (cps) mm^−2^.

### 2.2. 3D PCD imaging studies

To demonstrate the spatial resolution benefits of the PCD for 3D tomographic imaging, the PCD was operated under a UHR CBCT acquisition mode and reconstructed with a truncated ramp kernel with a cutoff frequency of 7 mm^−1^. Both PCD-CBCT and EID-CBCT images for comparison were acquired using a commercial C-arm system (Artis Zee, Siemens Healthineers) using pulsed x-rays under matched beam collimation (2.5 cm at iso) and radiation dose conditions (7.1 mGy CTDI_vol_), corresponding to a flux rate of approximately 7 × 10^7^ cps mm^−2^. CSC was turned off for PCD-CBCT acquisitions to reduce pulse pile-up-induced count losses since the PCD dead time is larger with CSC on than with CSC off (Ji *et al* 2019), and an almost order of magnitude higher x-ray flux was used for CBCT than the 2D acquisitions. No anti-scatter grid was used in these acquisitions since the narrow beam collimation of 2.5 cm limited the amount of scattered radiation to within acceptable levels for the high-contrast imaging task. Both EID- and PCD-CBCT images had a matched reconstruction voxel size of 0.07 mm, and EID-CBCT images were directly reconstructed by the commercial C-arm system with the highest resolution kernel available (“EE/Sharp” reconstruction). To compare detector technologies, UHR PCD-CT images were acquired with both the native unbinned pixels (100 *μ*m pitch) for the highest possible resolution, as well as 2 × 2 binning (200 *μ*m pitch) to closely match the native unbinned pixel size of the EID (194 *μ*m pitch). Due to limitations on the EID readout speed, unbinned EID-CBCT images can only be acquired using a longer scan time of 20 s compared to the 7 s protocol used for all other CBCT acquisitions in this work; therefore, EID-CBCT images with 2 × 2 binning (388 *μ*m pitch) were also acquired to compare to UHR PCD-CT images using the same gantry rotation speed. PCD-CBCT and EID-CBCT images of the 3 mm stent were acquired, and 3D reformatted images were then generated.

To demonstrate the benefit of PCD-CBCT in imaging small perforating blood vessels, *in vivo* animal experiments were performed under the approval of the University of Wisconsin-Madison Institutional Animal Care and Use Committee (IACUC). The subjects were two Yorkshire domestic swine with an approximate mass of 40 kg; iodinated contrast (Omnique 300, 60 ml, 5 ml s^−1^) was administered intravenously followed by 10 ml of saline flush using a power injector. A ‘Normal’ PCD-CBCT imaging mode was used, in which the PCD pixels were 4 × 4 binned, the reconstruction voxel size was set to 0.47 mm, and the reconstruction kernel was adjusted such that the system’s modulation transfer function (MTF) matched that of the EID-CBCT under the ‘HU/Normal’ mode with 2 × 2 binning of the native EID pixels (Ji *et al* 2021). Axial MIPs were generated over a range of 4.2 mm, and contrast-to-noise ratios (CNR) of five large iodinated blood vessels were measured in the MIP images.

PCD- and EID-CBCT acquisitions used a 7 s rotation time, with 494 projection views that cover an angular span of 200° (180° plus fan angle) using a clinical CBCT scan protocol programmed into the C-arm, and were reconstructed with a conventional FBP algorithm with the Parker short scan weighting. Geometric distortions of the C-arm gantry rotation were found to be reproducible from scan to scan, and were calibrated with a single gantry rotation using a BB helix phantom to generate view-by-view projection matrices that were incorporated during FBP (Ji *et al* 2021).

### 2.3. PCD-EID image fusion for full FOV coverage

In order for the integrated PCD-EID concept to be viable, the functionality of the detector assembly for full FOV imaging must remain intact. As shown in figure 9, simply combining the PCD and EID images result in noticeable boundaries in the resulting images where the PCD meets the EID due to differences in image quality from the two detection mechanisms such as signal, contrast, and image texture: the different photon energy-weightings between PCDs and EIDs inherent to their detection mechanisms result in measured x-ray attenuations at different effective energies, and thus generally different signal and contrast; additionally the different conversion mechanisms and detector pixel sizes result in spatial resolution and noise texture differences. Therefore, the PCD images must be post-processed to match the characteristics of the EID image to avoid artifactual boundaries that may interfere with IGIs by degrading image quality when a full FOV is needed, and when the advantages of the PCD are not required.

**Figure 2.**
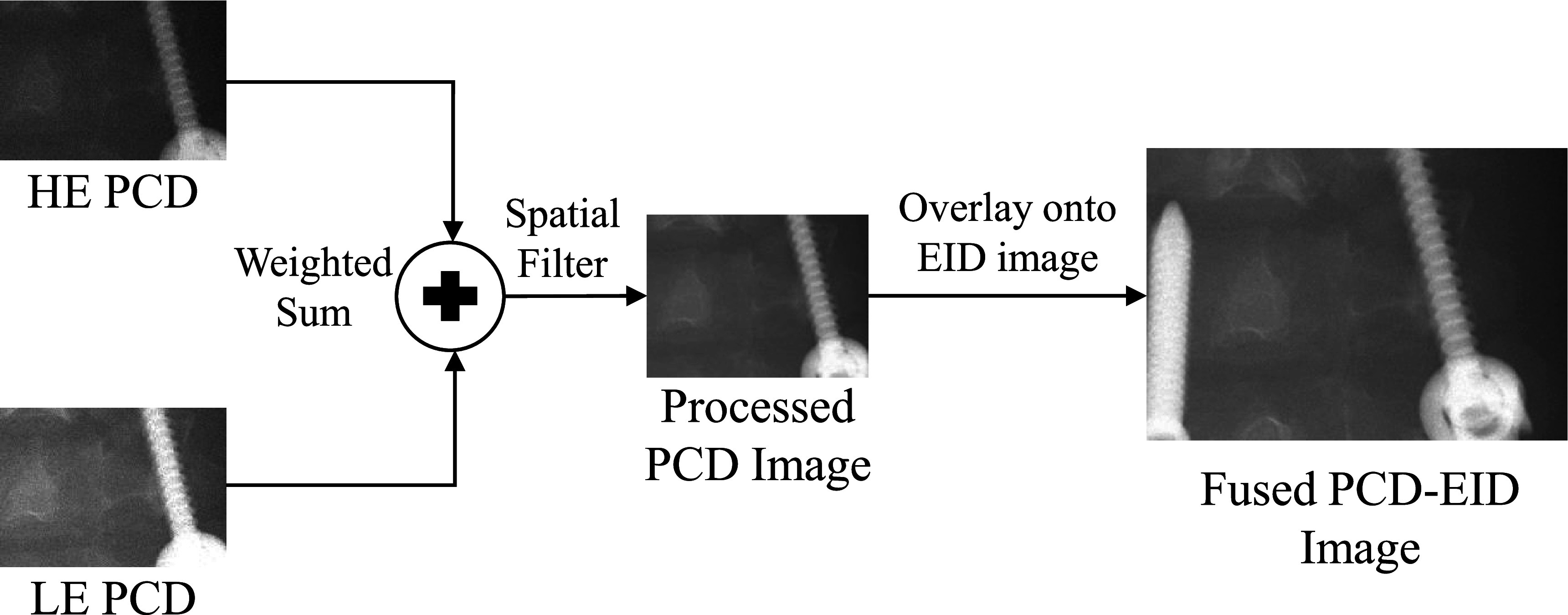
Schematic of the PCD post-processing pipeline to fuse the central PCD image with the surrounding EID image. HE: high-energy bin; LE: low-energy bin.

As a proof-of-concept for PCD-EID image quality matching, post-log 2D PCD images were post-processed in two steps as shown in figure 2:

(i) To match the signal and contrast of the EID images, PCD images were generated using an empirical weighted sum of the post-log HE and LE PCD images to match the measured x-ray attenuation of the EID.
(ii) An empirical Gaussian filter with a standard deviation of 150 *μ*m was applied to the new signal-matched PCD image to match its noise texture and spatial resolution to the EID, which contains less high-frequency components than the PCD due to spatial blurring in the scintillator and the larger detector pixel size.

For the signal and contrast matching, the post-log HE and LE PCD images were combined as

\begin{eqnarray*}{\mathrm{PCD}}_{\mathrm{weighted}}=(a\times \mathrm{HE})+(b\times \mathrm{LE}),\end{eqnarray*}

where PCD_weighted_ is the PCD image with matched signal to the EID image, and *a* and *b* are free parameters with the constraint that *a* + *b* = 1. As a first approximation, *a* and *b* are chosen such that the average pixel value in the weighted PCD image was equal to the average pixel value in the EID image for the same ROI. The parameters are then fine-tuned so that the weighted PCD image matches the EID image visually as closely as possible. The resulting post-processed PCD images were then overlaid onto the EID images to generate an integrated PCD-EID image free of boundaries and artifacts. Note that due to the difference between the source-to-detector distances in the prototype imaging system used in this work, the PCD images had to be magnified slightly so that they could be registered with the EID images of the same object prior to image fusion. To validate this post-processing method, experimental PCD and EID images of a BB helix phantom and a titanium pedicle screw (Synthes 6.2 mm × 35 mm Titanium) that was inserted near the cervical spine of an anthropomorphic head phantom (ACS CT Head Phantom, Kyoto Kagaku Co., Ltd, Japan) were acquired at 120 kV and an air Kerma per images of 0.11 *μ*Gy (x-ray flux rate of approximately 9 × 10^6^ cps mm^−2^).

## 3. Experimental results

### 3.1. 2D imaging results

**Figure 3.**
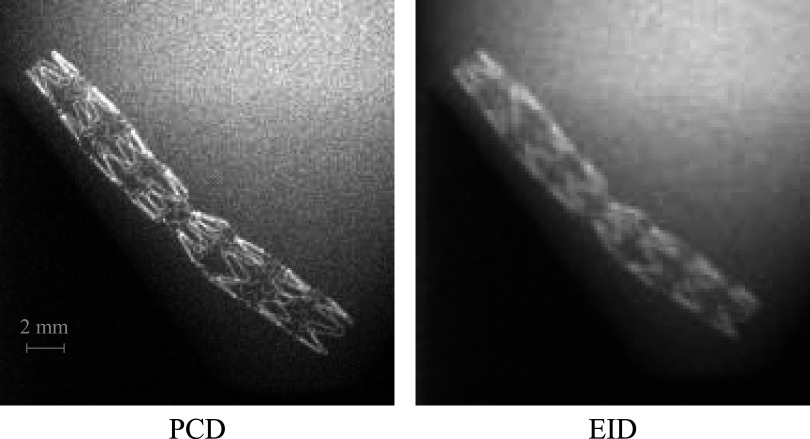
Region-of-interest 2D PCD and EID images of the 3 mm stent demonstrating the spatial resolution benefits of the PCD. The pure EID image is displayed to compare to the PCD image rather than displaying a fused PCD-EID image.

**Figure 4.**
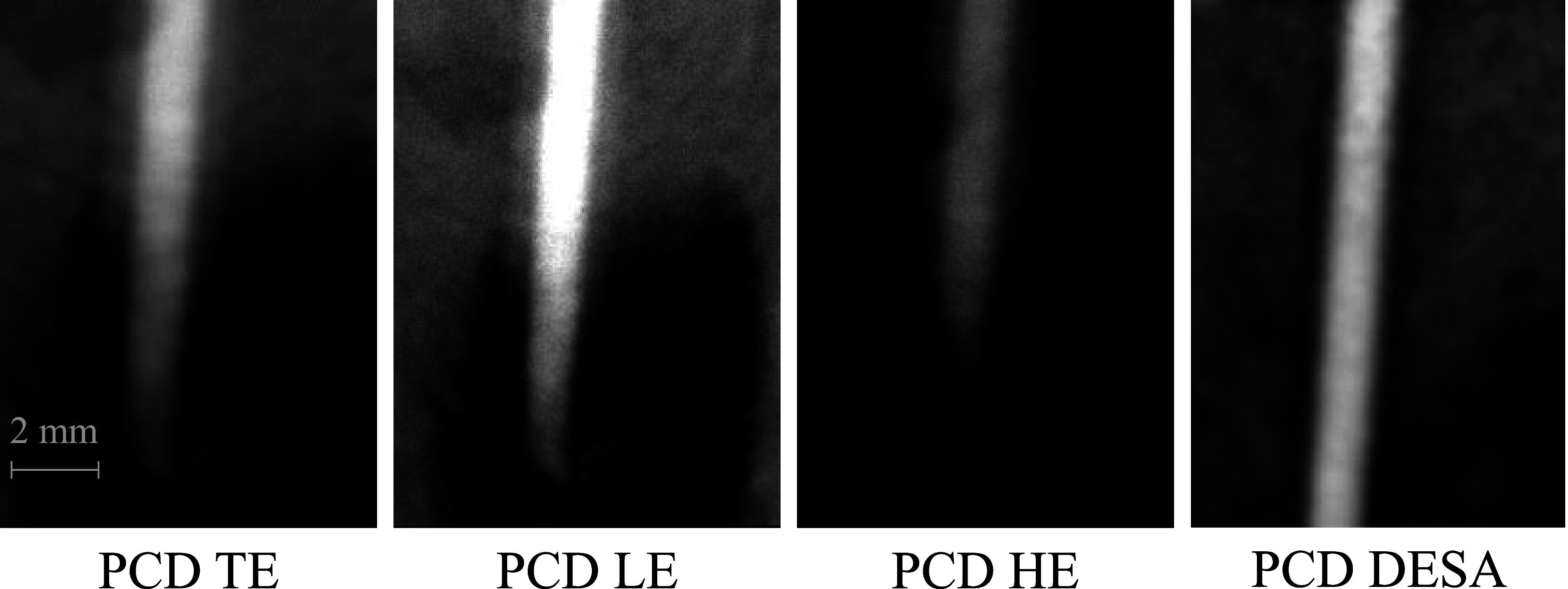
Region-of-interest 2D PCD images of the iodinated vessel phantom. TE: total-energy bin; LE: low-energy bin; HE: high-energy bin; DESA: dual-energy subtraction angiography.

Figure 3 shows 2D ROI images of the stent acquired with the PCD module and the EID module both using their native unbinned pixel sizes. The PCD image shows the fine stent wires with less spatial blurring due to the direct-conversion sensor and the smaller available PCD pixels. Notably, the thinner wires in the narrower ‘kinked’ section of the stent can be delineated by the PCD, but are unresolved by the EID. Figure 4 shows total-energy bin (TE), low-energy bin (LE), high-energy bin (HE), and DESA PCD images of the iodinated vessel phantom highlighting a region of artifactual stenosis caused by overlaying tissue in the TE image. The DESA successfully removes the overlaying tissue signal by combining the information from the LE and HE images to clearly delineate the entire vessel in this region. This subtraction of the overlaying tissue signal is similar to DSA but eliminates the possibility of misregistration artifacts since the LE and HE PCD images are acquired simultaneously.

### 3.2. 3D imaging results

**Figure 5.**
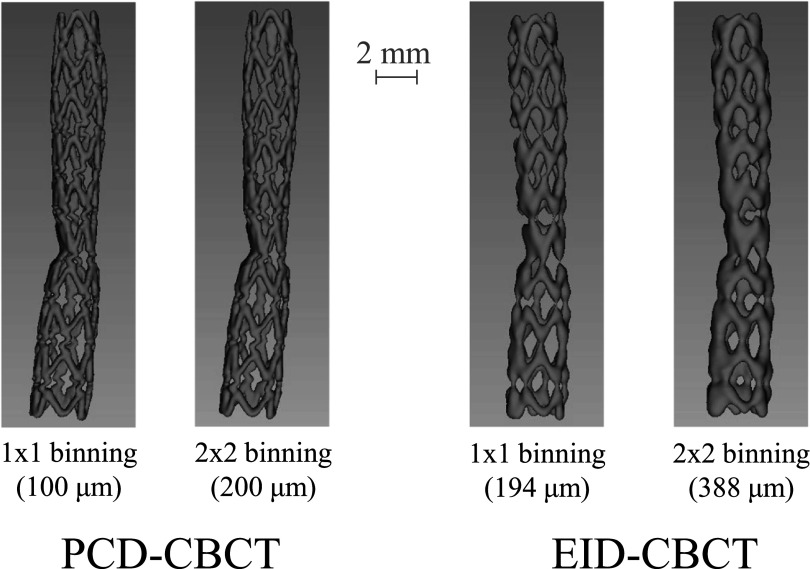
UHR PCD-CBCT and EID-CBCT 3D reformatted images of the 3 mm stent under different detector pixel binning conditions. The PCD-CBCT demonstrates improved delineation of the stent wires even when the PCD pixels are binned to match the pitch of the unbinned EID pixels. The pure EID images are displayed to compare to the PCD image rather than displaying a fused PCD-EID image.

**Figure 6.**
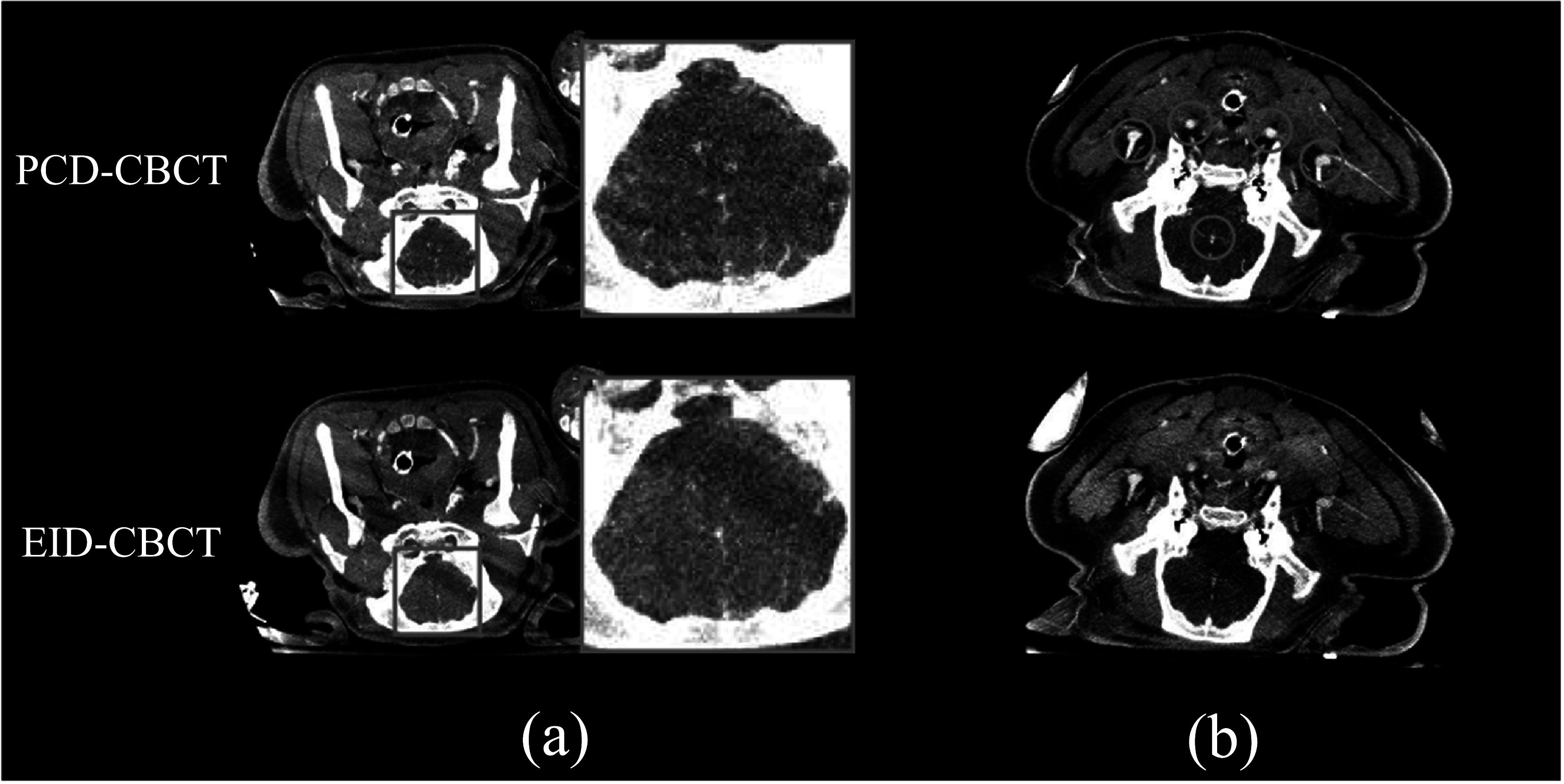
PCD-CBCT and EID-CBCT MIP images of an *in vivo* swine subject demonstrating (a) improved visualization of small peripheral vessels and (b) improved CNR in the five indicated vessels in the PCD-CT image. The pure EID images are displayed to compare to the PCD image rather than displaying a fused PCD-EID image. Image display range: [−100 to 300 HU].

The UHR PCD-CBCT 3D reformatted images in figure 5 show improved delineation of the stent wires compared to the EID-CBCT images even when the PCD pixels are 2 × 2 binned to match the detector pixel size of the EID. Again, note that the unbinned (1 × 1) EID-CBCT was acquired under a longer 20 s rotation rather than a 7 s rotation due to detector readout speed limitations when the pixels are unbinned. These images are similar to those obtained with a more narrow PCD-CT in previous work (Treb *et al* 2022) but can be obtained with a single gantry rotation rather than with the multiple rotations needed to obtain sufficient z-coverage in the previous work. For the *in vivo* animal study, figure 6 shows PCD-CBCT and EID-CBCT MIPs of the brain region of two swine subjects covering the entire axial cross-section. Figure 6(a) shows several small cerebral vessels that are visible in the PCD-CBCT image but are missed by the EID-CBCT. This result is consistent with previous literature on the benefits of PCDs for CTA (Harvey *et al* 2019), and is due to the more optimal photon energy-weighting of the PCD as well as improved spatial resolution potential over the EID. The average CNR over the five larger vessels in figure 6(b) was measured to be 8.3 (range: (6.2, 10.3)) in the PCD-CT images and 4.2 (range: (3.3, 5.4)) in the EID-CBCT images, resulting in an average CNR improvement of 103% (range: (29%, 151%)).

### 3.3. PCD-EID image fusion

**Figure 7.**
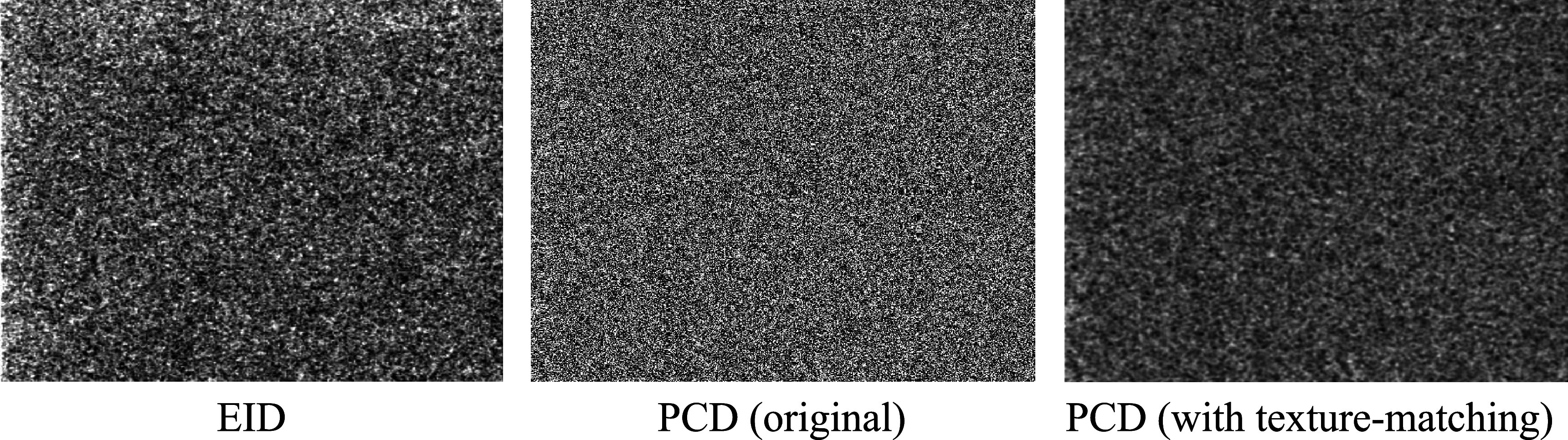
Noise texture comparison between 2D EID and PCD images without and with the proposed post-processing. The display window is matched across all images.

**Figure 8.**
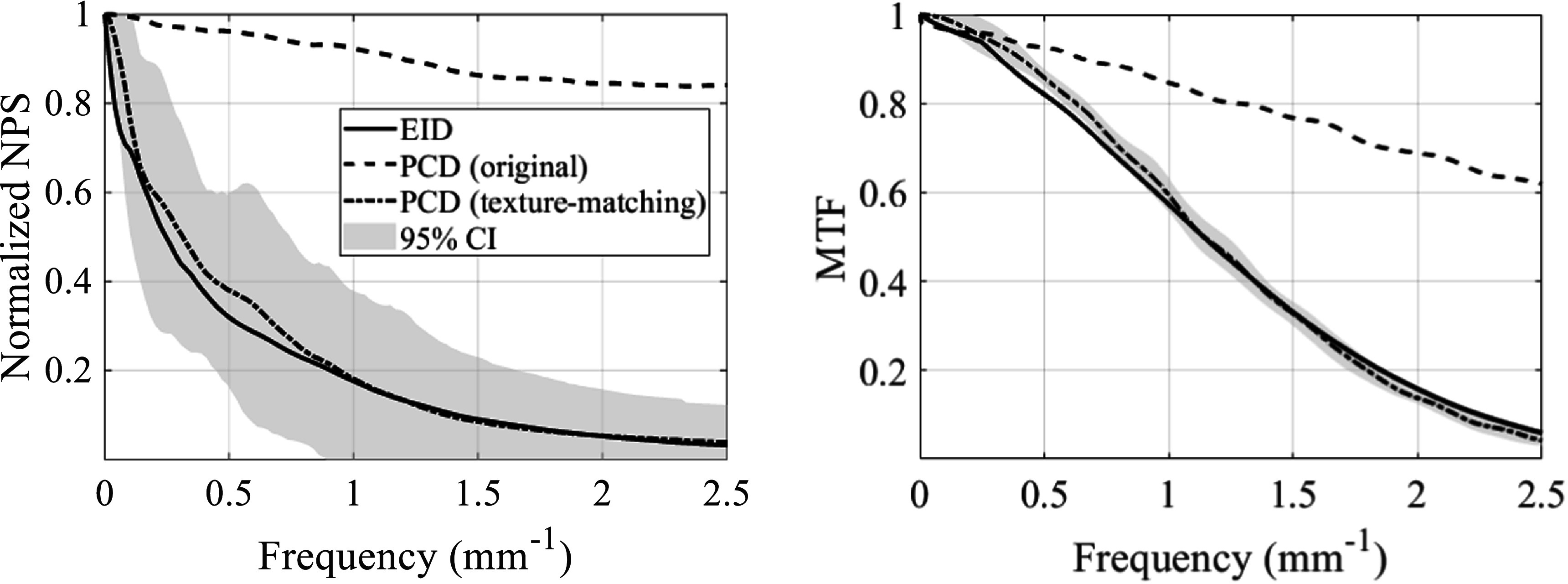
Radially averaged normalized noise power spectra (NPS), and modulation transfer functions (MTFs) of 2D EID and PCD images (before and after the proposed post-processing). CI: confidence interval.

For full FOV imaging using both the central PCD and the surrounding EID simultaneously, the noise texture of the original unprocessed PCD image is shown in figure 7 side-by-side with an EID image and the PCD image with the proposed post-processing. Due to the sharper spatial resolution of the PCD compared to the EID, its original noise texture has more prominent high-frequency components than the EID image. After the spatial filter is applied, this high-frequency noise in the PCD image is suppressed, resulting in a similar noise texture to the EID to enable seamless integration of the two detector outputs to form a single full FOV image. Note that the noise magnitude may be different between the EID and post-processed PCD in general using this method, since the goal is to match noise *texture* rather than *magnitude*. Repeated single image acquisitions were performed for each detector, and noise power spectra (NPS) were calculated from the EID and pre- and post-filtered PCD image sequences; the radial average of the 2D normalized NPS displayed up to the Nyquist frequency of the EID are shown in figure 8 to compare their shapes, along with 95% confidence intervals calculated from the variance of the NPS at each frequency across different radial directions for the texture-matched PCD image. The NPS shapes confirm that the noise texture of the filtered PCD image closely matches that of the EID image. Averaged MTFs and 95% confidence intervals were also calculated from the edge spread functions (ESFs) measured from eight independent ROIs in an image of a tungsten edge phantom; the tungsten edge was angled at 3 degrees from the perpendicular of the measurement direction to increase the spatial sampling density of the ESF (Samei *et al* 1998). Figure 8 demonstrates that the spatial filtering of the PCD image results in similar spatial resolution between the EID and PCD up to the Nyquist frequency of the EID. Note that for the purpose of removing obvious boundary artifacts between the PCD and EID images for an integrated PCD-EID detector, the NPS and MTFs do not need to be *exactly* matched, rather, they only need to be close enough so that the images from the two detectors are indistinguishable in the eyes of treating physicians.

**Figure 9.**
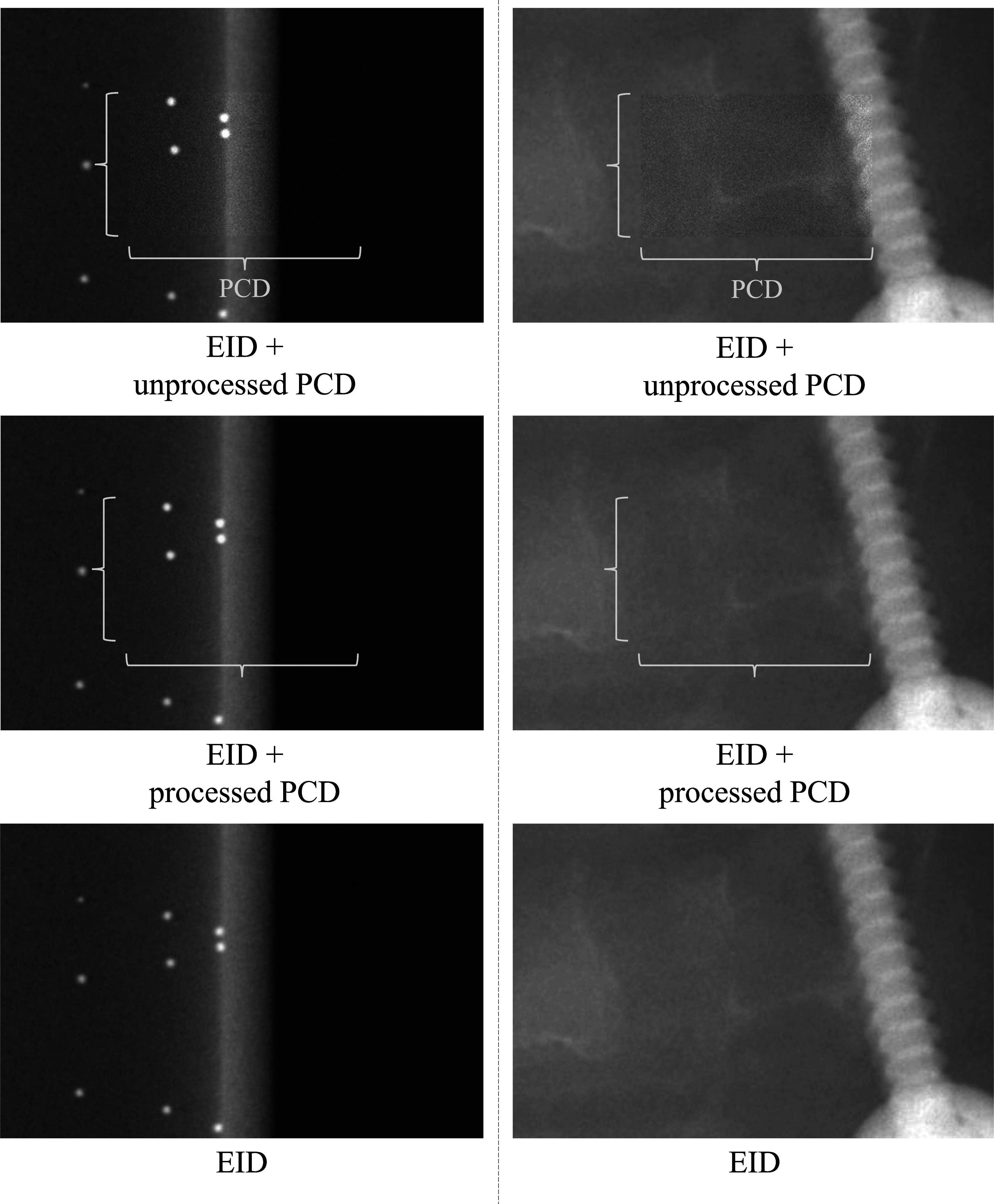
PCD images overlaid onto a flat panel EID image background of (left) a BB helix phantom, and (right) a pedicle screw near the cervical spine of a head phantom before and after the two-step post-processing. The displayed images are cropped from the full FOV for easier viewing. Original EID images with no PCD overlay are also displayed for comparison.

When the PCD signal/contrast matching is implemented using the LE and HE energy bins and followed by the spatial filtering, the PCD image of the BB helix phantom is seamlessly integrated into the EID image by mitigating differences in both image signal and texture (figure 9, left); when the unprocessed PCD image is overlaid onto the EID image, boundaries are present due to the differences in these image characteristics. Note that the displayed images in figure 9 are cropped to enlarge the central regions of the EID and the PCD for easier viewing. For the BB helix phantom, the weighting factors *a* and *b* in equation (2) were 0.61 and 0.39, respectively. The right column of figure 9 shows another case with the pedicle screw in the head phantom, with similar results to the BB images both before and after PCD post-processing: Boundaries are visible due to a generally different image signal brightness as well as background noise texture and spatial resolution differences at the edge of the screw. For these images, the weighting factors *a* and *b* in equation (2) were 0.73 and 0.27, respectively. The two-step PCD post-processing is simple and fast computationally (∼5 ms/frame) for potential implementation in real-time during dynamic imaging sequences with frame rates at or above 30 fps (the maximum frame rate of the current EID).

## 4. Discussion

Experimental imaging studies in this work using a prototype PCD module and an EID flat panel demonstrated the imaging benefits of the PCD-EID design for IGIs in both phantom and *in vivo* studies. The central PCD module can provide DESA capabilities as well as improved spatial resolution for UHR imaging in both 2D and 3D applications, and PCD-CBCT demonstrated improved visibility of small cerebral vessels over the EID-CBCT due to improvements in iodine CNR and spatial resolution. The PCD image can be integrated with a surrounding EID image after minimal post-processing of the PCD image to create a fused image that maintains the full FOV imaging capabilities of current FPDs for less demanding imaging sequences that do not require the additional imaging benefits offered by the PCD.

Previous work by our group involved the initial implementation of a narrow-width PCD prototype with 6 mm z-coverage with a commercial C-arm system for PCD-CT applications (Ji *et al* 2021), with emphasis on system engineering and artifact correction: Initial results demonstrated the potential of the prototype C-arm PCD-CT in reducing image noise compared to the EID-CBCT at matched spatial resolution, and image quality was improved by the PCD-CT over the EID-CBCT for low-contrast tasks such as for periprocedural ICH monitoring (Treb *et al* 2021). Additionally, the energy bins of the PCD were leveraged for CT image-domain material decomposition to discriminate between iodine and calcium. These previous results are directly applicable to the PCD module of the hybrid detector design. Subsequent works upgraded the same narrow-width PCD-CT C-arm system with step-and-shoot capabilities to enable volumetric imaging even with narrow beam collimation to avoid scatter and cone-beam artifacts (Treb *et al* 2022), and explored the high-resolution potential of the PCD for UHR PCD-CT. The hybrid PCD-EID design reported in this work represents the next step in developing this prototype PCD C-arm system, and introduces a wider PCD with 2.5 cm z-coverage to enable 2D PCD imaging and reduce the number of step-and-shoot gantry rotations for PCD-CBCT with a given z-coverage. This work also introduces a framework to combine the PCD and EID images into a single image.

Several alternative approaches to improve the imaging utility of C-arms for IGIs have been investigated in the literature: First, a 30 cm × 5 cm PCD has been mounted to a commercial C-arm system (Ahmad *et al* 2016, Müller *et al* 2016, Ahmad *et al* 2017). This approach demonstrated utility in dual-energy material decomposition as well as improved spatial resolution. However, the potential benefits of PCD for low-contrast CT imaging were not investigated in these studies. In contrast, the narrow-beam PCD-CT has experimentally demonstrated improved low-contrast visibility over the EID-CBCT in our previous work. Additionally, the problem of obtaining a uniform response over the entire PCD area reported in a previous study (Ahmad *et al* 2017) has been overcome through improved detector calibrations (Feng *et al* 2021). Other than PCDs, dual-layer energy-integrating FPDs can potentially introduce dual-energy imaging capabilities to commercial C-arms (Lu *et al* 2019, Ma *et al* 2020, Shi *et al* 2020b, 2020a), although this approach requires a filter material (e.g. copper) between the two detector layers for adequate spectral separation (Cai *et al* 2023), and x-ray absorption in this filter layer may reduce the detection efficiency and noise performance of dual-layer FPDs for non-spectral imaging sequences. Fast-kV switching can also introduce dual-energy imaging to C-arms (Müller *et al* 2016, Speidel *et al* 2019, Nikolau *et al* 2022), but offers no additional benefits for non-spectral applications, and furthermore may result in misregistration artifacts caused by patient motion during the transition time between the two tube potentials. Lastly, a more cost-effective approach than using PCDs is to replace the a-Si:H transistors in EIDs with CMOS (Jain *et al* 2015, Sheth *et al* 2018, Job *et al* 2019, Abiola *et al* 2020, Sheth *et al* 2020) to reduce electronic noise, improve spatial resolution, reduce lag, and increase the maximum frame rate.

The experimental studies performed in this work present a proof-of-concept for the hybrid PCD-EID flat panel design and its clinical utility, but the preliminary nature of the experiments gives rise to several limitations. First, the PCD and EID acquisitions were done separately, and the two detector technologies have not yet been combined into a single detector piece. The preliminary results in this work have paved the way for a truly integrated single-piece hybrid PCD-EID flat panel that will be developed for future studies. For example, for the experimental proof-of-concept image acquisitions in this work the PCD was mounted in front of the EID, but for the proposed integrated PCD-EID flat panel design the PCD module is slightly behind the EID module. Therefore, the magnification of the PCD subsystem would be different between the prototype presented in this work and the proposed truly integrated PCD-EID, which will exacerbate the impact of focal spot blurring compared to our preliminary imaging experiments. Therefore, careful consideration and optimization of system magnification, detector pixel size and binning, and focal spot size are important in the implementation of the proposed PCD-EID flat panel. Another important consideration is how a truly integrated PCD-EID flat panel may affect automatic exposure rate control (AERC) performance, although we do not expect the added PCD module to significantly impact the AERC since the AERC cell (typically a flat ion chamber) is typically placed in front of the detector, and both the CdTe used in the PCD and the CsI used in the EID are high-Z materials with negligible backscatter towards the AERC cell.

Additionally, the high frame-rate potential of PCDs (up to 1000 fps) was not investigated for dynamic imaging sequences such as fluoroscopy and DSA, rather, static images were acquired to demonstrate the advantages of the PCD over the EID. Other works have demonstrated the potential of high-frame-rate PCD for angiographic imaging (Krebs *et al* 2019, Nagesh *et al* 2022a, 2022b, Troville *et al* 2022, Williams *et al* 2022, Wu *et al* 2022), and our future work will follow in these footsteps to further characterize the benefits of PCDs for interventional imaging applications. Furthermore, the proof-of-concept image quality matching between the PCD and EID in this work was only done with static post-log images rather than pre-log images or dynamic image sequences such as those that would be acquired during conventional fluoroscopy, and the weighting factors for the LE and HE images to match the EID signal and contrast were empirically determined. For eventual clinical translation, the image quality matching must be done for all possible full FOV imaging sequences (both post- and pre-log) as well as for different x-ray spectra and object sizes and compositions, and the matching must be done automatically without any user intervention. This represents an additional challenge since the weighting factors *a* and *b* in equation (2) were observed to have a dependence on the image object size, e.g. the head phantom required a higher weighting of the HE PCD image than the BB helix phantom, since the head phantom is more attenuating and thus hardens the x-ray beam more than the smaller helix phantom. We expect parameters *a* and *b* to have a similar dependence on the kV and x-ray beam filtration used. These limitations call for additional innovations in image post-processing and fusion, which is a topic of our future research. Lastly, the presented DESA PCD images used a simple Gaussian filter to reduce the amplified image noise in the dual-energy subtraction: other denoising approaches such as anti-correlated noise reduction (McCollough *et al* 1989) or non-linear approaches (Hinshaw and Dobbins 1995) may also be utilized.

## 5. Conclusions

A hybrid flat-panel detector design utilizing a cost-effective central PCD integrated with a surrounding large-area scintillator-based EID can potentially upgrade C-arm interventional x-ray systems with spectral and dose-efficient ultra-high resolution capabilities without interfering with the clinical need for full FOV imaging.

## Data Availability

The data cannot be made publicly available upon publication because no suitable repository exists for hosting data in this field of study. The data that support the findings of this study are available upon reasonable request from the authors.
